# Integrating memory-guided saccades and EEG for MCI screening: a multimodal LASSO modeling approach

**DOI:** 10.3389/fnagi.2026.1821547

**Published:** 2026-05-28

**Authors:** Xinying Zhao, Fuda Yu, Hui Wang, Yanling Kang, Yiting Zhu, Zhongfeng Xiao, Donger Bai, Jiamin Li

**Affiliations:** Department of Neurology, Shijiazhuang People’s Hospital, Shijiazhuang, China

**Keywords:** early biomarker, electroencephalography, LASSO regression, memory-guided saccades, mild cognitive impairment

## Abstract

**Background:**

We investigated differences in memory-guided saccades (MGS) and electroencephalographic (EEG) spectral ratios between cognitively normal older adults and patients with mild cognitive impairment (MCI). A multimodal predictive model was developed to evaluate its potential for early MCI identification.

**Methods:**

We enrolled 60 participants (37 MCI, 23 controls) who underwent cognitive assessments, 32-channel resting-state EEG, and MGS testing. EEG spectral ratios, including the Delta-to-Alpha Ratio (DAR), Theta-to-Alpha Ratio (TAR), and (Delta+Theta)/(Alpha+Beta) Ratio (DTABR), were extracted along with MGS parameters (latency, accuracy, gain). To prevent data leakage, 21 multimodal features were evaluated using a zero-leakage least absolute shrinkage and selection operator (LASSO) pipeline. A multivariable predictive model was subsequently constructed using Firth’s penalized logistic regression, explicitly adjusting for demographic covariates, and internally validated with 1,000 bootstrap resamples.

**Results:**

MCI patients exhibited significantly prolonged bilateral saccadic latencies, decreased accuracy, and widespread cortical spectral slowing (elevated DAR, TAR, and DTABR across all regions) (all *P* < 0.05). The LASSO model identified a seven-feature cross-modal diagnostic panel. Following Firth’s penalization, rightward saccadic accuracy maintained its significance as an independent protective factor. The combined nomogram achieved an apparent AUC of 0.994 and a bootstrap optimism-corrected AUC of 0.975, demonstrating favorable calibration and promising net clinical benefit.

**Conclusion:**

MCI patients demonstrate coupled oculomotor and electrophysiological abnormalities, reflecting impaired frontoparietal-sensorimotor network efficiency. Our rigorously penalized multimodal model yields robust internal diagnostic performance, providing a promising exploratory proof-of-concept for the early screening of “cognitive-motor decoupling” in clinical neurology, though large-scale external validation is warranted.

## 1 Introduction

With population aging accelerating worldwide, the prevalence of cognitive disorders has risen substantially on a global scale. The World Health Organization estimates that approximately 78 million individuals will be living with dementia by 2030, increasing to 139 million by 2050 (Gustavsson et al., 2023). Cognitive impairment, referring to a subjective or objective decline in cognitive domains such as memory, comprehension, reasoning, and attention, has emerged as a major public health concern threatening the health and independence of older adults. Mild cognitive impairment (MCI) represents a critical transitional stage between normal cognitive aging and early dementia. It is primarily characterized by measurable cognitive decline, most commonly in memory—while basic activities of daily living remain largely preserved (Albert et al., 2011; Petersen et al., 2018). Epidemiological data indicate that the prevalence of MCI among individuals aged over 60 years ranges from 6.7 to 25.2%, with an annual conversion rate to dementia of approximately 5–17% (Ward et al., 2012; Petersen et al., 2018). In the context of rapid population aging, early identification and timely intervention during the MCI stage are of paramount importance in delaying further neurodegenerative progression.

At present, the clinical diagnosis of MCI relies predominantly on cognitive screening instruments in conjunction with clinical history. Commonly used tools in routine practice include the Mini-Mental State Examination (MMSE) (Folstein et al., 1975) and the Montreal Cognitive Assessment (MoCA) (Nasreddine et al., 2005). While widely adopted due to their accessibility and ability to quantify global cognitive performance, these behavior-based assessments rely heavily on structured questioning. Consequently, scale-derived scores do not directly reflect the underlying central neuropathological processes driving cognitive decline. In the earliest stages of impairment, performance on a single screening measure may be heavily influenced by educational attainment and cognitive reserve, potentially masking subtle deficits (Jannati et al., 2024). Although structural and functional neuroimaging, as well as cerebrospinal fluid biomarkers, provide high pathological specificity, their high cost, invasiveness, and requirement for specialized facilities limit their feasibility for large-scale community screening. There remains a pressing need for objective, non-invasive, and cost-effective tools for early detection.

Emerging evidence suggests that early functional decline in the central nervous system is frequently accompanied by measurable abnormalities in oculomotor control (Lage et al., 2020). Alterations in eye movement patterns directly reflect impairments in visuospatial memory, episodic memory, and executive function, all of which are hallmark features of early cognitive decline (Kahana Levy et al., 2018). Compared with traditional questionnaire-based approaches, eye-tracking paradigms offer quantifiable and rater-independent measurements. Among these, memory-guided saccades (MGS) constitute a complex visuocognitive paradigm that robustly engages working memory, attentional allocation, and voluntary motor output, making it an optimal behavioral probe for early network degeneration (Rane et al., 2023).

Parallel to behavioral metrics, electroencephalography (EEG) offers a high-temporal-resolution window into cortical network dynamics. Beyond conventional absolute power metrics, quantitative EEG (qEEG) spectral analysis has increasingly utilized specific frequency ratios—including the Delta-to-Alpha Ratio (DAR), Theta-to-Alpha Ratio (TAR), and (Delta+Theta)/(Alpha+Beta) Ratio (DTABR)—to characterize early cortical dysrhythmia (Moretti, 2015). However, a critical appraisal of these spectral approaches reveals both significant pathophysiological insights and practical challenges. On the one hand, these markers demonstrate high sensitivity to the pathological “spectral slowing” characteristic of early neurodegeneration, often capturing thalamocortical uncoupling and synaptic dysfunction before measurable deficits manifest on standard cognitive scales (D’Atri et al., 2021). On the other hand, despite their high physiological sensitivity, the widespread clinical utility of individual qEEG markers is occasionally hampered by inter-individual heterogeneity and a lack of standardized recording protocols across diverse cohorts (Geng et al., 2022; Miraglia et al., 2023). Both impairments in MGS performance and EEG spectral slowing have been closely linked to the degeneration of frontoparietal circuits, a core neuropathological substrate of early MCI. To date, however, these two complementary modalities have not been systematically integrated to evaluate clinical MCI.

In the present study, we integrated the MGS paradigm with qEEG spectral analysis to investigate differences in oculomotor parameters and electrophysiological power spectra between cognitively normal older adults and individuals with clinical MCI. Through this multimodal approach, we aimed to establish an exploratory, non-invasive screening framework. By addressing the limitations of single-modality assessments, this study serves as an exploratory physiological proof-of-concept for the early identification of “cognitive-motor” decoupling in MCI.

## 2 Materials and methods

### 2.1 Participants

Between July 2024 and August 2025, a total of 60 inpatients were consecutively recruited from the Department of Neurology at Shijiazhuang People’s Hospital. Demographic and clinical data were collected for all participants, including age, sex, years of education, history of hypertension, fasting blood glucose, low-density lipoprotein (LDL), plasma homocysteine (Hcy), and the extent of White Matter Hyperintensities (WMHs) on brain MRI.

Participants were classified into a mild cognitive impairment (MCI) group and a normal cognition (NC) group according to predefined inclusion and exclusion criteria.

#### 2.1.1 Inclusion criteria–normal cognition group

(1) Age ≥ 50 years; (2) No subjective or objective cognitive complaints; (3) Cognitive function within the normal range as confirmed by standardized screening tests, defined as MMSE ≥ 27 and MoCA ≥ 26; (4) Activities of daily living fully preserved, with no evidence of functional impairment;(5) Adequate visual acuity to complete the memory-guided saccades; (6) No history of epilepsy, Parkinson’s disease, major psychiatric disorders, or other neurological conditions affecting cognition.

#### 2.1.2 Inclusion criteria–MCI group

(1) Age ≥ 50 years; (2) Diagnosis consistent with the International Classification of Diseases, 11th Revision (ICD-11) criteria for mild neurocognitive disorder (code 6D70) (Gaebel et al., 2018; Gaebel et al., 2019), characterized by subjective and/or objective cognitive decline with largely preserved activities of daily living; (3) Mild cognitive impairment confirmed by standardized cognitive screening, defined as MMSE < 27 and/or MoCA < 26; (4) Visual acuity sufficient to perform the MGS, either uncorrected or corrected to normal; (5) No history of epilepsy, Parkinson’s disease, major psychiatric disorders, or other neurological conditions affecting cognition.

#### 2.1.3 Exclusion criteria

Severe visual impairment precluding participation in visual tasks; Cognitive impairment severe enough to significantly interfere with social or occupational functioning and meeting diagnostic criteria for dementia (Gaebel et al., 2019; First et al., 2023); Unstable psychiatric illness or current treatment for major psychiatric disorders; Use of antidepressants, antipsychotics, or other medications known to significantly affect cognitive or neural function; Inability to complete the MGS or EEG procedures.

This study was conducted in accordance with the Declaration of Helsinki and approved by the Ethics Committee of Shijiazhuang People’s Hospital (Approval No. 2025-119). Written informed consent was obtained from all participants and their legal representatives prior to enrollment.

### 2.2 Cognitive assessment

All participants completed the Mini-Mental State Examination (MMSE) and the Montreal Cognitive Assessment (MoCA) in a quiet, well-lit environment. Assessments were administered independently by trained professionals following standardized procedures (Pendlebury et al., 2013).

The MMSE is widely used for cognitive screening in older adults due to its simplicity and ease of administration. The total score is 30 points, with a commonly used cutoff of 27; scores below 27 suggest possible cognitive impairment (Folstein et al., 1975). The MoCA demonstrates greater sensitivity than the MMSE in detecting deficits in executive function, attention, memory, and visuospatial abilities. In this study, the Beijing version of the MoCA was administered (total score 30). A score ≥ 26 was considered cognitively normal. In accordance with standard recommendations, one additional point was added for participants with fewer than 12 years of formal education to adjust for educational bias (Nasreddine et al., 2005).

### 2.3 Memory-guided saccades and EEG recording

**FIGURE 1:**
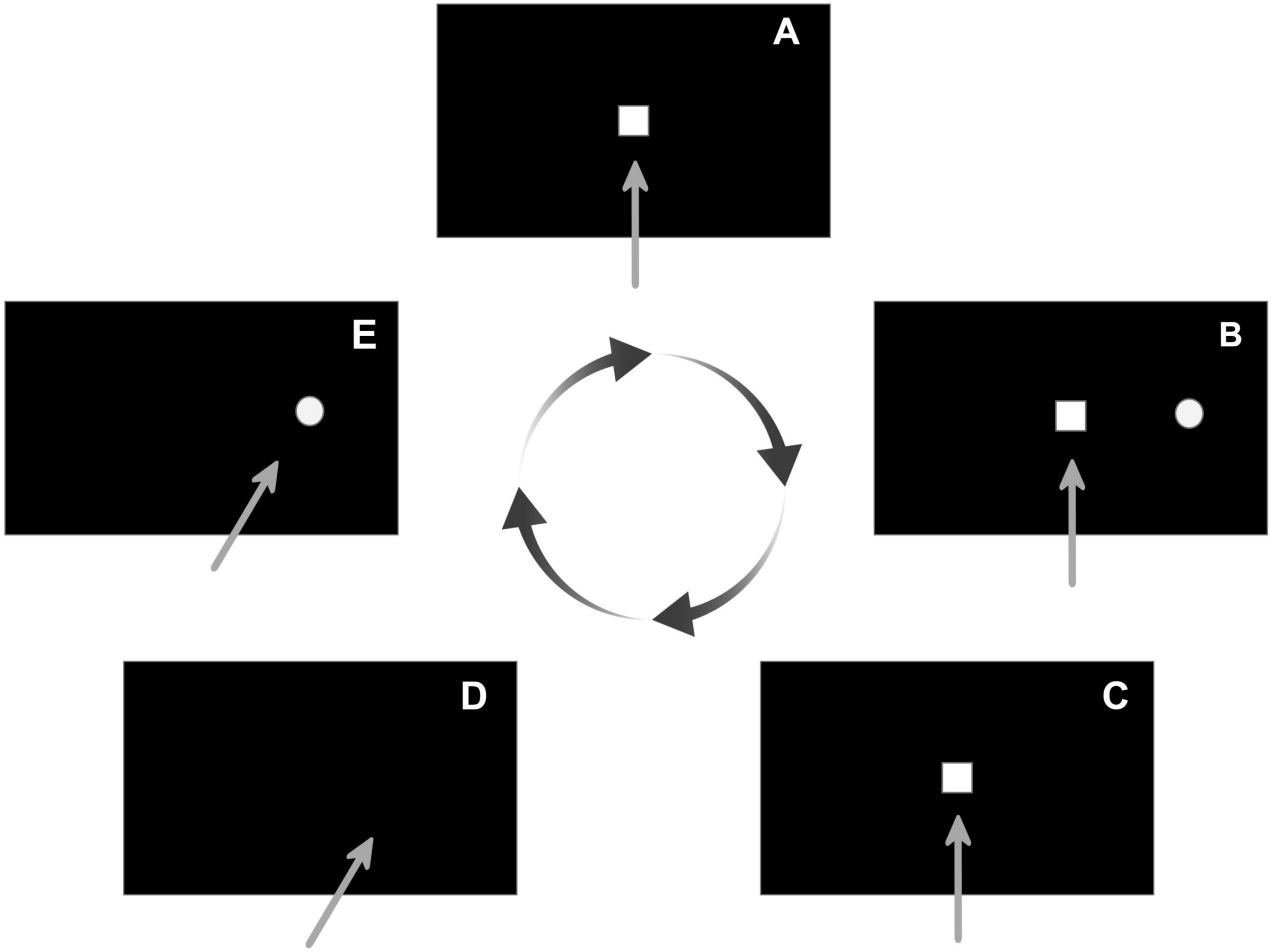
Five stages of the memory-guided saccades (MGS) paradigm **(A–E)**.

All experiments were conducted in a sound-attenuated, softly illuminated, electromagnetically shielded room. Participants were seated comfortably at a viewing distance of approximately 1 meter from the display screen. Continuous 32-channel video electroencephalography (Neurofax EEG-1200, Nihon Kohden) was recorded using electrodes placed according to the international 10–20 system. Reference electrodes were positioned on both earlobes. Signals were sampled at 512 Hz. Electrode impedance was maintained below 5 kΩ. Sensitivity was set at 10 μV/mm, and the paper speed equivalent was 30 mm/s. During EEG acquisition, participants were instructed to remain awake, relaxed, and seated quietly with eyes closed during the resting-state recording, while minimizing blinking and body movements. All unnecessary electronic devices were switched off to reduce potential interference. Resting-state EEG was recorded for 5 min, after which participants proceeded to the memory-guided saccades (Leng et al., 2024). The MGS paradigm was presented using VertiPACS software. The experimental sequence was as follows: A. Central fixation: A fixation point appeared at the center of the screen for 800 ms; B. Peripheral target flash: While central fixation remained on the screen, a peripheral target was briefly presented in a random direction for 200 ms; C. Delay period: After target disappearance, a 300 ms delay interval followed; D. Saccade execution: At the end of the delay period, the central fixation point disappeared, serving as the “go” cue. Participants were instructed to shift their gaze as quickly and accurately as possible to the remembered target location; E. Target fixation: Participants were required to fixate the target location as rapidly and precisely as possible. The MGS lasted 210 s, after which EEG recording was terminated (Figure 1).

### 2.4 Data preprocessing

All EEG data were preprocessed and analyzed using MATLAB R2024b. Raw EEG recordings were imported and processed as follows: Re-referencing: Signals were re-referenced to the average of the bilateral earlobes. Filtering: A band-pass filter (0.5–70 Hz) was applied to remove low-frequency drift and high-frequency electromyographic noise. A 50 Hz notch filter was additionally implemented to eliminate power line interference. Artifact rejection: Two experienced neurologists independently performed visual inspection to identify and exclude artifact-contaminated segments, including those affected by prominent muscle activity, eye movement artifacts, or poor electrode contact. Segmentation: For each participant, a continuous 30-s artifact-free resting-state EEG segment was selected for subsequent analysis. The selection of a 30-s epoch represents a necessary methodological trade-off in an elderly cohort; while longer continuous recordings can theoretically reduce spectral variance, a strict 30-s window ensures signal stationarity by minimizing the risk of vigilance fluctuations (e.g., micro-sleeps) and motion artifacts, providing sufficiently stable power estimates for standard frequency bands.

Fast Fourier Transform (FFT) was applied to convert EEG signals into the frequency domain. In accordance with the International Federation of Clinical Neurophysiology (IFCN) guidelines (Kane et al., 2017) and to ensure mutually exclusive frequency bins during digital computation, the spectral bands were mathematically defined with strictly non-overlapping boundaries: delta (δ, 0.5–3.9 Hz), theta (θ, 4.0–7.9 Hz), alpha (α, 8.0–12.9 Hz), and beta (β, 13.0–30.0 Hz) (Cao et al., 2022). The gamma band (>30.0 Hz) was deliberately excluded from the current analysis due to its well-documented susceptibility to cranial electromyographic (EMG) artifact contamination in older adults, which could introduce substantial noise into our 30-s stationary epoch analyses (Goncharova et al., 2003; Muthukumaraswamy, 2013). To minimize interindividual variability in total power, relative power (RP) was calculated for each band as the proportion of power within a specific frequency band relative to the total power across all bands. Based on the international 10–20 system, electrodes were grouped into five regions of interest (ROIs): frontal (F3, F4, Fz), central (Cz), parietal (P3, P4, Pz), occipital (O1, O2), and temporal (T3, T4). The mean relative power for each ROI was calculated as the arithmetic average of all electrodes within that region. Three commonly used spectral ratios were subsequently computed to characterize the distribution of low- and high-frequency activity: DAR = Pδ/Pα; TAR = Pθ/Pα; DTABR = (Pδ + Pθ)/(Pα + Pβ). Where Pδ, Pθ, Pα, and Pβ represent the mean relative power of the corresponding frequency bands. All ratios were calculated at the ROI level and averaged for statistical analysis.

Raw eye-tracking data from all MGS trials underwent quality control procedures, including assessment of missing values and outliers. The following MGS parameters were extracted: Accuracy: Defined as the proportion of valid trials in which the saccade endpoint fell within the predefined target area, reflecting task performance correctness. Latency: The interval (in milliseconds) between central fixation offset and saccade initiation, indexing response speed and executive control. Final saccadic gain: A measure of endpoint precision, defined as the ratio of the distance between the saccade endpoint and the target location relative to the intended amplitude. A gain of 100% indicates perfect alignment with the target; gain > 100% indicates overshoot; gain < 100% indicates undershoot (Ettinger et al., 2003; Ettinger et al., 2005).

### 2.5 Statistical analysis

All statistical analyses were performed using SPSS version 25.0 and R software (version 4.5.2). Continuous variables were tested for normality using the Kolmogorov–Smirnov test. Normally distributed variables are presented as mean ± standard deviation and were compared between groups using independent-samples *t*-tests. Non-normally distributed variables are presented as median (P25, P75) and were compared using the Mann–Whitney U test. Categorical variables are expressed as counts and percentages and were analyzed using the chi-square test or Fisher’s exact test, as appropriate. For correlation analyses between EEG spectral measures and MGS parameters, Pearson or Spearman correlation coefficients were applied according to the data distribution. To strictly control for Type I errors arising from multiple comparisons, the Benjamini-Hochberg False Discovery Rate (FDR) correction was applied to all univariate analyses (pooled across all EEG and MGS parameters). Statistical significance was defined as an FDR-adjusted *q*-value < 0.05.

To develop the multivariable prediction model, we adhered to the Transparent Reporting of a multivariable prediction model for Individual Prognosis or Diagnosis (TRIPOD) reporting guidelines. To strictly prevent data leakage and optimism bias during high-dimensional feature selection, no univariate pre-screening was performed, and a rigorous zero-leakage validation pipeline was implemented. Specifically, right−skewed EEG spectral ratios underwent natural logarithmic transformation, and all continuous candidate variables were Z-score standardized strictly within the cross-validation and bootstrap resampling loops. To address the challenge of high-dimensional feature selection and prevent model overfitting in our clinical cohort, we implemented the Least Absolute Shrinkage and Selection Operator (LASSO) regression. The LASSO estimator$\hat{\beta }$ is defined as the solution to the following optimization problem:


$$\hat{\beta }=\arg\,\min_{\beta }\left\{\overset{n}{\underset{i=1}{\sum }}(y_{i}-\overset{p}{\underset{j=1}{\sum }}x_{\mathrm{ij}}\,\beta_{j}{)}^{2}+\lambda \,\overset{p}{\underset{j=1}{\sum }}\left|\beta_{j}\right|\right\}$$

Where *y*_*i*_ represents the binary diagnostic label (MCI vs. NC), *x*_*ij*_ denotes the *j*-th neurophysiological feature for the *i*-th participant, β_*j*_ represents the regression coefficient, and λ is the regularization parameter. The *L*_1_ penalty term, $\lambda \,\sum_{j=1}^{p}\left|\beta_{j}\right|$, shrinks the coefficients of non-contributory variables exactly to zero, thereby achieving simultaneous feature selection and regularization.

Our implementation strategy followed a multi-stage validation pipeline: (1) Preprocessing: All candidate variables underwent Z-score standardization strictly within each cross-validation fold to prevent data leakage. (2) Hyperparameter Optimization: Ten-fold cross-validation was employed to determine the optimal penalty parameter (λ). The one-standard-error rule (λ_*1se*_) was applied to promote maximum model sparsity and reduce overfitting. (3) Stability Assessment and Internal Validation: A bootstrap resampling procedure with 1,000 iterations was performed to estimate the average optimism bias and to record the selection frequency of each feature, thereby rigorously evaluating model stability.

Following feature selection, a Firth’s penalized logistic regression was employed using the logistf package in R to rigorously address potential parameter estimation bias, data separation issues, and the numerical class imbalance between the MCI and NC groups. This method ensures robust and consistent estimates even in small-sample scenarios where standard maximum likelihood estimation may fail. By penalizing the maximum likelihood function with a Jeffreys invariant prior, this approach mitigates small-sample bias and yields finite, reliable estimates of odds ratios (ORs) and their 95% confidence intervals (CIs). Crucially, within this Firth’s multivariable regression model, we adjusted for predefined demographic covariates (age and education level) to isolate the independent predictive value of the core multimodal features against confounding clinical factors.

To facilitate clinical interpretation, a nomogram was constructed based on the final integrated model. Model discrimination was quantified using the area under the receiver operating characteristic curve (AUC). Calibration was visually assessed via a 1,000-bootstrap resampling calibration curve to evaluate the agreement between predicted and observed probabilities. Finally, Decision Curve Analysis (DCA) was performed to determine the net clinical benefit of the predictive model across a continuum of high-risk threshold probabilities, thereby evaluating its practical utility in clinical settings.

## 3 Results

### 3.1 Baseline characteristics

According to the predefined inclusion and exclusion criteria, 37 participants were included in the MCI group and 23 in the NC group. There were no statistically significant differences between groups in sex, age, years of education, history of hypertension, LDL, Hcy, fasting glucose, or the degree of white matter hyperintensities (WMHs) on MRI (all *P* > 0.05), indicating comparability in demographic and metabolic characteristics (Table 1).

**TABLE 1:** Baseline characteristics of the NC and MCI groups.

Variable	NC (*n* = 23)	MCI (*n* = 37)	χ^2^/Z	*P*-value
Gender [n (%)]			0.776	0.379
Male	11 (47.8%)	22 (59.5%)		
Female	12 (52.2%)	15 (40.5%)		
Age (years)	59.5 (55.0, 67.0)	62.2 (59.0, 68.5)	−1.218	0.223
Hypertension [n (%)]			0.406	0.524
Yes	15 (65.2%)	27 (73.0%)		
No	8 (34.8%)	10 (27.0%)		
LDL-C (mmoL/L)	2.96 (2.56, 3.32)	2.74 (2.22, 3.11)	−1.833	0.067
Hcy (μmoL/L)	15.50 (11.50, 15.90)	16.94 (10.90, 18.50)	−1.255	0.209
Fasting glucose (mmoL/L)	5.90 (5.30, 5.80)	6.05 (5.40, 6.85)	−1.317	0.188
Education level [n (%)]			2.141	0.143
Junior high school or below	14 (60.9%)	29 (78.4%)		
High school or above	9 (39.1%)	8 (21.6%)		
White matter hyperintensities [n (%)]			3.319	0.134
Grade 0–1	22 (95.7%)	29 (78.4%)		
Grade 2–3	1 (4.3%)	8 (21.6%)		

NC, Normal Cognition; MCI, Mild Cognitive Impairment; LDL-C, Low-Density Lipoprotein Cholesterol; Hcy, Homocysteine. Continuous variables are presented as median (interquartile range, P25–P75) and analyzed using the Mann-Whitney U test (yielding *Z*-values). Categorical variables are presented as counts (percentages) and analyzed using the Chi-square test (yielding χ^2^ values).

### 3.2 Differences in memory-guided saccades performance

**FIGURE 2:**
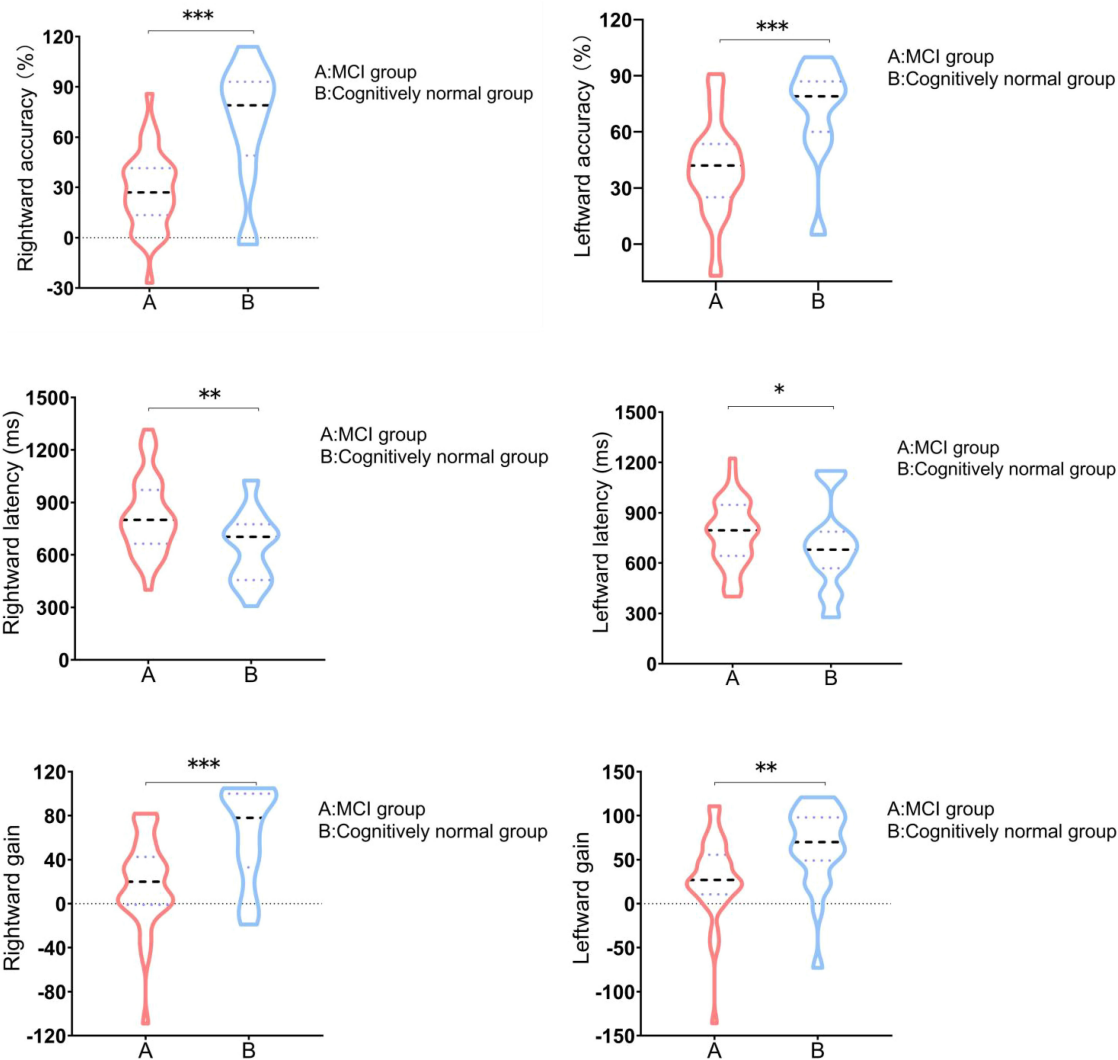
Distribution of MGS parameters in the NC and MCI groups. The violin plots illustrate the comparison of leftward and rightward saccadic parameters between the MCI group (A, red) and the cognitively normal group (B, blue). *P < 0.05, **P < 0.01, and ***P < 0.001 compared with the NC group.

During the MGS task, the MCI group demonstrated significantly prolonged saccadic latency in both the leftward and rightward visual fields (both FDR-adjusted *q* < 0.05). Concurrently, saccadic accuracy and final saccadic gain were significantly reduced in the MCI group compared to the NC group (all FDR-adjusted *q* < 0.05) (Figure 2 and Table 2).

**TABLE 2:** MGS Performance between the NC and MCI groups.

Variable	NC (*n* = 23)	MCI (*n* = 37)	Z	*Q*-value
Leftward accuracy (%)	72.74 (60.00, 87.00)	39.78 (25.00, 53.50)	−4.290	<0.001
Leftward latency (ms)	691.30 (569.00, 787.00)	808.19 (660.00, 952.00)	−2.235	0.025
Leftward gain	65.39 (49.00, 98.00)	28.89 (10.50, 55.50)	−3.224	0.001
Rightward accuracy (%)	69.09 (49.00, 93.00)	27.81 (13.50, 41.50)	−4.198	<0.001
Rightward latency (ms)	624.35 (439.00, 775.00)	804.89 (663.50, 971.00)	−2.783	0.005
Rightward gain	62.39 (33.00, 100.00)	18.43 (−1.00, 42.50)	−3.551	<0.001

MGS, Memory-Guided Saccades; NC, Normal Cognition; MCI, Mild Cognitive Impairment. Data are presented as median (interquartile range, P25–P75). The *q*-values represent statistical significance after the Benjamini-Hochberg False Discovery Rate (FDR) correction for multiple comparisons (pooled across all 21 univariate tests in this Tables 2, 3).

### 3.3 Differences in EEG spectral features

**FIGURE 3:**
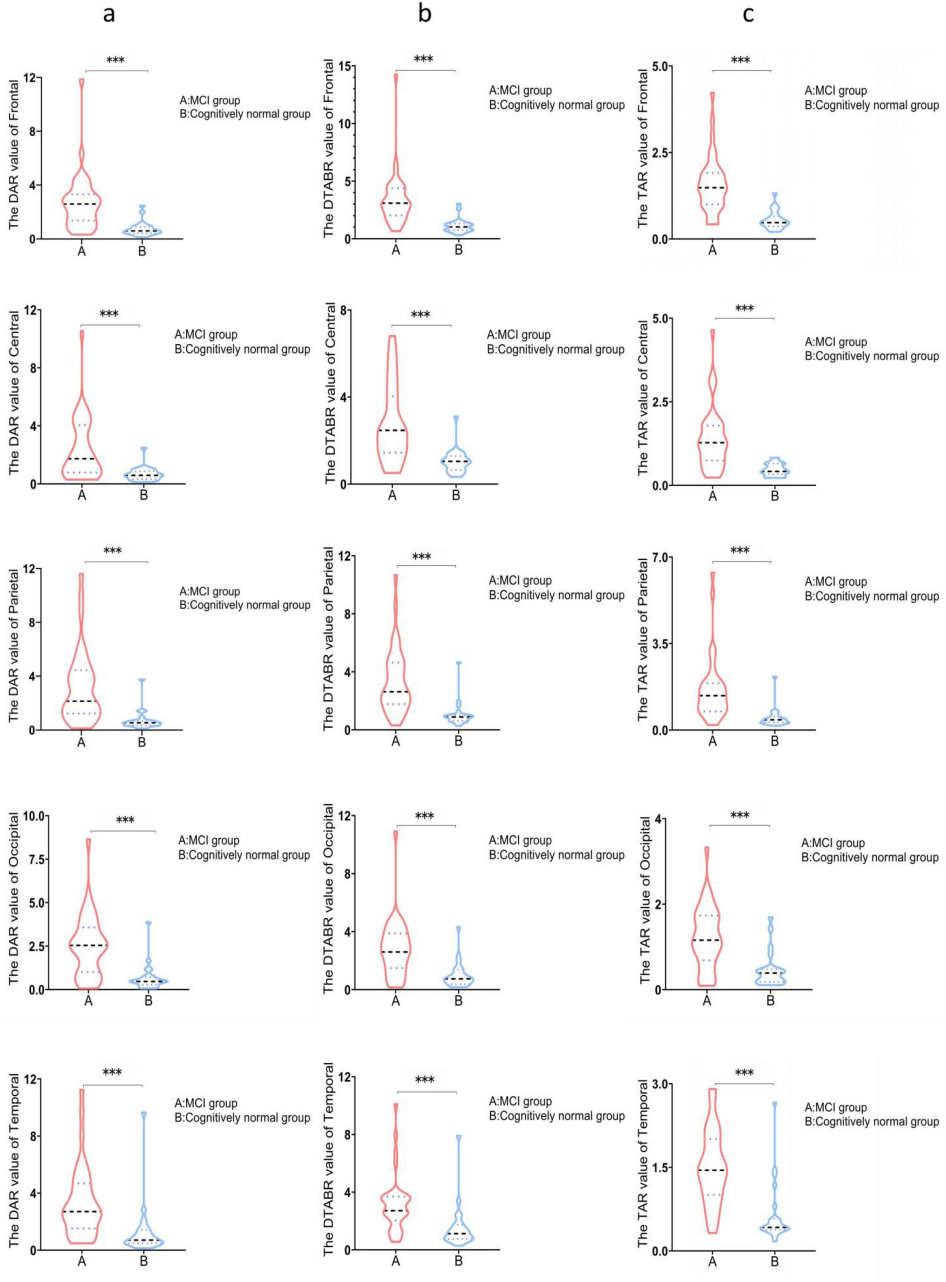
Distribution of EEG spectral ratios across five cortical regions in the NC and MCI groups. The figure illustrates the comparison of **(a)** DAR, **(b)** DTABR, and **(c)** TAR values across the frontal, central, parietal, occipital, and temporal regions between the MCI group (A, red) and the NC group (B, blue). DAR, Delta-to-Alpha Ratio; DTABR, (Delta+Theta)/(Alpha+Beta) Ratio; TAR, Theta-to-Alpha Ratio. ***P < 0.001 compared with the NC group.

Across all five regions of interest —frontal, central, parietal, occipital, and temporal, the MCI group exhibited significantly higher DAR, TAR, and DTABR values compared with the NC group. These neurophysiological differences remained highly significant even after rigorous Benjamini-Hochberg correction for multiple comparisons (all FDR-adjusted *q* < 0.05) (Figure 3 and Table 3).

**TABLE 3:** Comparison of EEG ratio parameters between the NC and MCI groups.

Variable		NC (*n* = 23)	MCI (*n* = 37)	Z	*Q*-value
Frontal lobe	FDAR	0.7480 (0.4185, 0.8916)	2.7081 (1.3654, 3.3211)	−4.949	<0.001
	FDTABR	1.1295 (0.7401, 1.3208)	3.3612 (2.0300, 4.3925)	−5.375	<0.001
	FTAR	0.5490 (0.3618, 0.6563)	1.5890 (1.0004, 1.9094)	−5.481	<0.001
Parietal lobe	PDAR	0.7060 (0.3479, 0.6845)	3.2707 (1.2301, 4.4487)	−4.843	<0.001
	PDTABR	1.0490 (0.6166, 1.0120)	3.3517 (1.7771, 4.6356)	−5.010	<0.001
	PTAR	0.4906 (0.3104, 0.5440)	1.6360 (0.7545, 1.9004)	−4.995	<0.001
Occipital lobe	ODAR	0.6851 (0.2782, 0.7117)	2.5220 (1.0104, 3.5727)	−4.394	<0.001
	ODTABR	0.9974 (0.3809, 1.3687)	2.8218 (1.4958, 3.8818)	−4.227	<0.001
	OTAR	0.4584 (0.1813, 0.4756)	1.2244 (0.6874, 1.7351)	−4.060	<0.001
Temporal lobe	TDAR	1.3044 (0.4850, 1.4215)	3.3641 (1.5236, 4.6846)	−4.349	<0.001
	TDTABR	1.5537 (0.7590, 1.7426)	3.1380 (2.0370, 3.6949)	−4.166	<0.001
	TTAR	0.6473 (0.3769, 0.5780)	1.4937 (1.0065, 2.0088)	−4.698	<0.001
Central	CDAR	0.6806 (0.3319, 0.8789)	2.3646 (0.7810, 4.0536)	−3.885	<0.001
	CDTABR	1.0467 (0.6464, 1.2883)	2.7907 (1.4424, 4.0379)	−4.523	<0.001
	CTAR	0.4747 (0.3333, 0.6503)	1.3869 (0.7435, 1.7901)	−4.858	<0.001

NC, Normal Cognition; MCI, Mild Cognitive Impairment; DAR, Delta-to-Alpha Ratio; DTABR, (Delta+Theta)/(Alpha+Beta) Ratio; TAR, Theta-to-Alpha Ratio. Data are presented as median (interquartile range, P25–P75). The *q*-values represent statistical significance after the Benjamini-Hochberg False Discovery Rate (FDR) correction for multiple comparisons (pooled across all 21 univariate tests in this Tables 2, 3).

### 3.4 Correlation analyses

**FIGURE 4:**
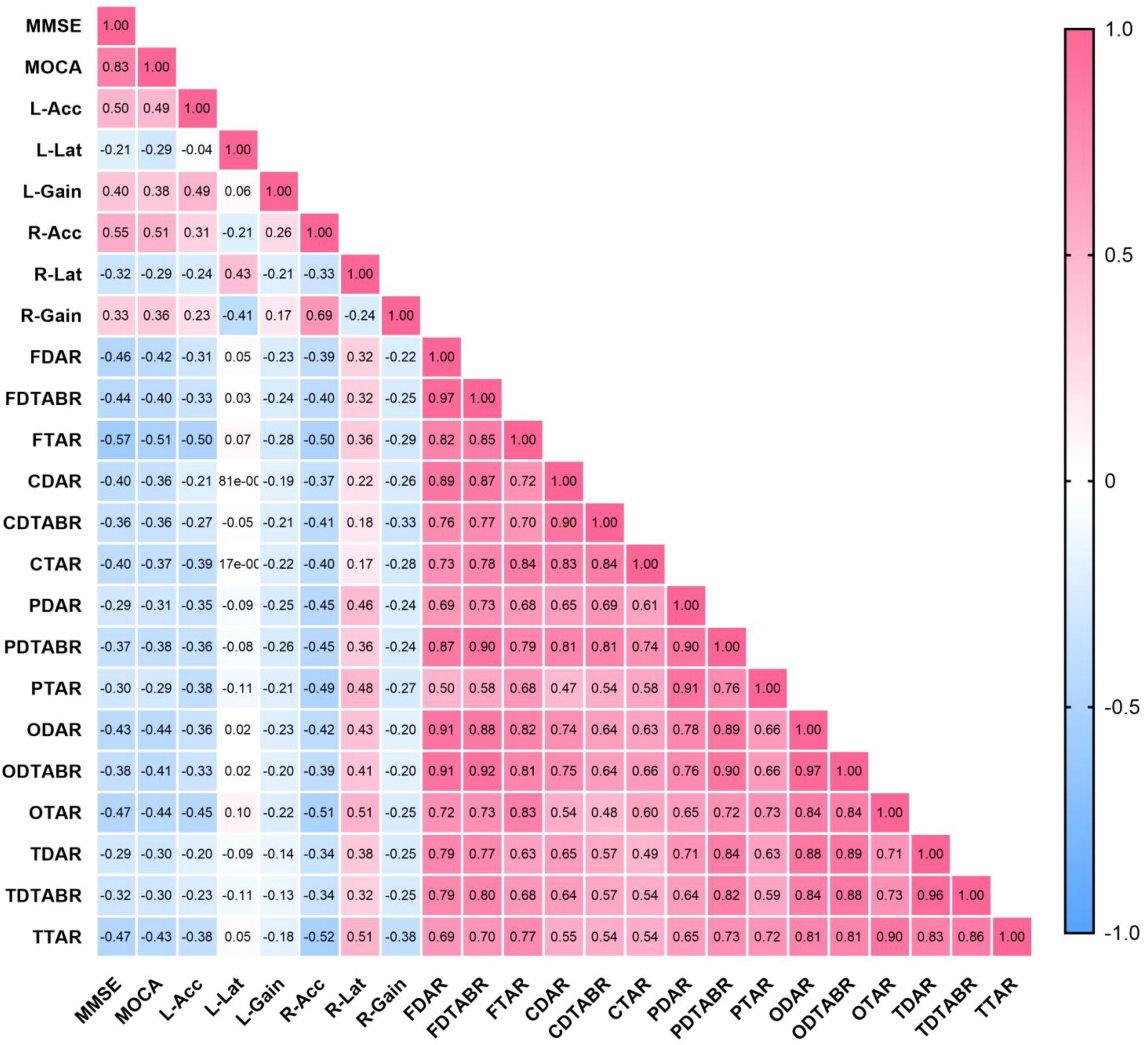
Correlation heatmap of cognitive scores, MGS parameters, and EEG spectral ratios. L-Acc, Leftward accuracy; L-Lat, Leftward latency; L-Gain, Leftward gain; R-Acc, Rightward accuracy; R-Lat, Rightward latency; R-Gain, Rightward gain; FDAR, Frontal Delta-to-Alpha Ratio; FDTABR, Frontal (Delta+Theta)/(Alpha+Beta) Ratio; FTAR, Frontal Theta-to-Alpha Ratio (similar nomenclature applies to other regional prefixes: C, Central; P, Parietal; O, Occipital; T, Temporal).

**FIGURE 5:**
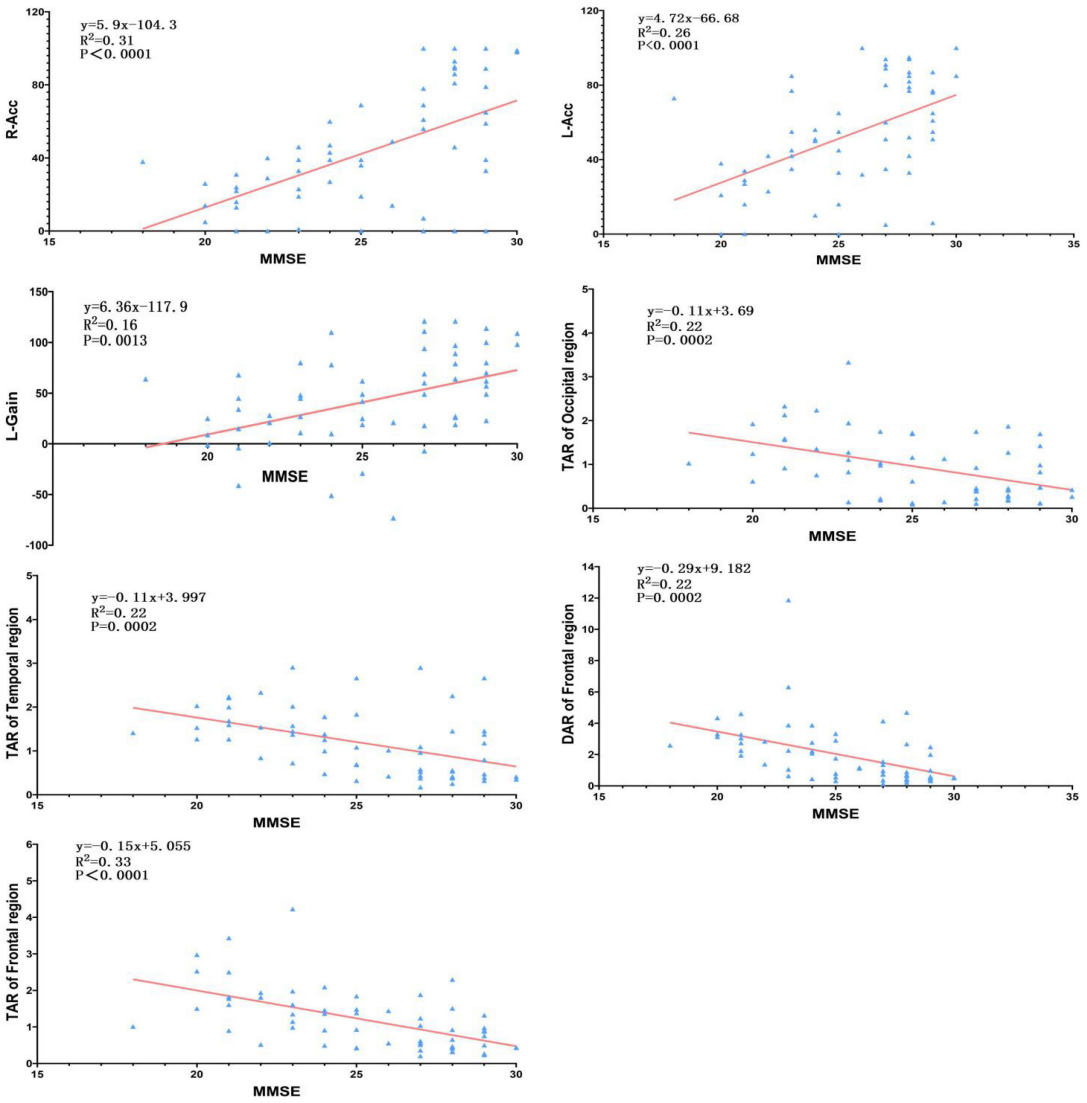
Regression fits between MMSE scores and selected MGS and EEG parameters. MMSE, Mini-Mental State Examination; R-Acc, Rightward accuracy; L-Acc, Leftward accuracy; L-Gain, Leftward gain; DAR, Delta-to-Alpha Ratio; TAR, Theta-to-Alpha Ratio.

MoCA and MMSE scores were significantly associated with both MGS parameters and EEG spectral ratios (including DTABR, TAR, and DAR). Specifically, MMSE and MoCA scores were positively correlated with leftward and rightward saccadic accuracy as well as leftward saccadic gain. Conversely, cognitive scores were negatively correlated with several spectral ratios, including frontal TAR, frontal DAR, temporal TAR, and occipital TAR. Further curve-fitting analyses indicated that selected MGS parameters and EEG spectral ratios demonstrated linear or near-linear relationships with cognitive scores (Figures 4, 5).

### 3.5 Development and validation of an MCI risk prediction model based on cross-modal features

**FIGURE 6:**
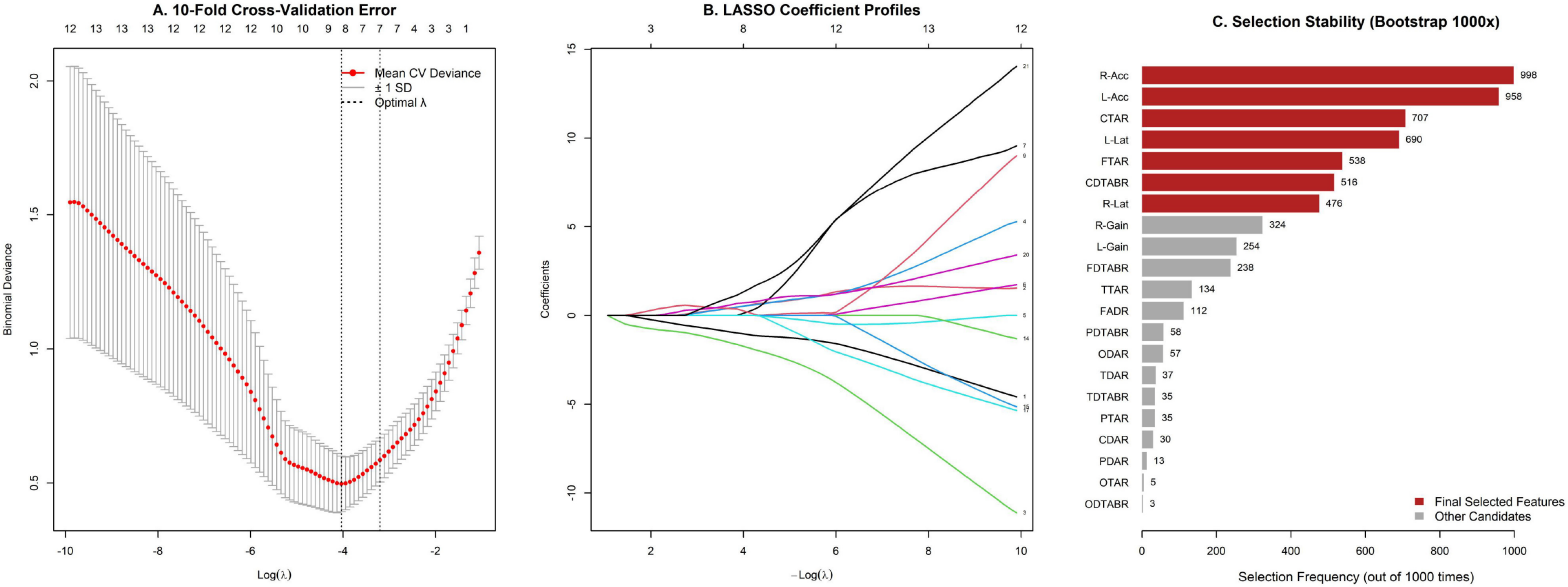
LASSO-based dimension reduction for cross-modal features and verification of resampling stability. This figure illustrates the process of screening and assessing the stability of key predictive features for mild cognitive impairment, based on LASSO regression and 1,000 bootstrap resamples. **(A)** LASSO 10-fold cross-validation error curve: The optimal regularization parameter (λ_1*se*_) was selected based on the one-standard-error criterion to obtain a relatively parsimonious and stable model. **(B)** LASSO coefficient path plot: Illustrates the shrinkage trajectories of the regression coefficients. At λ = λ_1*se*_, the regression coefficients of 14 variables were shrunk to zero, ultimately retaining 7 non-zero features. **(C)** Feature selection frequency analysis based on 1,000 bootstrap resamples: The red bar chart represents the 7 core features ultimately included in the multivariable model; the gray bar chart represents the unselected candidate variables. L-Acc, Leftward accuracy; R-Acc, Rightward accuracy; L-Lat, Leftward latency; R-Lat, Rightward latency; CTAR, Central Theta-to-Alpha Ratio; FTAR, Frontal Theta-to-Alpha Ratio; CDTABR, Central (Delta+Theta)/(Alpha+Beta) Ratio.

In this study, all 21 predefined cross-modal candidate variables were included in a LASSO regression model. Ten-fold cross-validation was used to estimate the model bias, and the optimal regularization parameter (λ_1*se*_) was selected on this basis. The regression coefficients of 14 variables were reduced to zero, leaving seven features with non-zero regression coefficients: leftward accuracy, rightward accuracy, leftward saccadic latency, rightward saccadic latency, frontal TAR, central DTABR, and central TAR. The stability of this feature selection was rigorously evaluated via 1,000 bootstrap resamples. As detailed in the updated Figure 6C, four core features demonstrated high selection stability ( > 60%), including rightward saccadic accuracy (99.8%), leftward accuracy (95.8%), central TAR (70.7%), and leftward latency (69.0%). Three features exhibited moderate stability: frontal TAR (53.8%), central DTABR (51.6%), and rightward saccadic latency (47.6%). Although rightward latency was sensitive to sample perturbations, it maintained a clear margin over the unselected candidates and was therefore retained to preserve the complementary integrity of the multimodal signature.

In the unadjusted analysis (Table 4, Model 1), rightward saccadic accuracy emerged as a significant protective factor against MCI (OR = 0.141, 95% CI: 0.003–0.569, *P* = 0.002). Subsequently, a fully adjusted model (Table 4, Model 2) was constructed to control for age and education level. Following stringent adjustment, rightward saccadic accuracy robustly maintained strict statistical significance as an independent protective factor (OR = 0.196, 95% CI: 0.007–0.975, *P* = 0.045). Furthermore, leftward saccadic accuracy demonstrated a strong protective trend (OR = 0.304, *P* = 0.076). Notably, the wide confidence intervals observed for certain EEG spectral ratios (e.g., central DTABR) in both models are indicative of quasi-complete separation, a phenomenon typical of high-dimensional electrophysiological data in relatively modest sample sizes.

**TABLE 4:** Multivariable logistic regression analysis of cross-modal predictors for MCI.

Cross-modal features	Model 1: unadjusted	*P*-value	Model 2: adjusted	*P*-value
	OR (95% CI)		OR (95% CI)	
Leftward accuracy	0.250 (0.011–1.297)	0.101	0.304 (0.001–1.143)	0.076
Rightward accuracy	0.141 (0.003–0.569)	0.002	0.196 (0.007–0.975)	0.045
Leftward latency	2.390 (0.764–34.476)	0.146	2.013 (0.626–22.301)	0.244
Rightward latency	1.908 (0.393–18.870)	0.413	1.545 (0.347–10.033)	0.577
FTAR	1.330 (0.140–10.697)	0.774	1.623 (0.155–11.978)	0.625
CDTABR	4.058 (0.076–95561.920)	0.574	3.591 (0.073–35305.216)	0.613
CTAR	1.501 (0.002–256.832)	0.870	1.405 (0.004–206.952)	0.900
Age	–	–	1.264 (0.318–22.139)	0.741
Education level	–	–	0.961 (0.014–5.297)	0.975

OR, Odds Ratio; CI, Confidence Interval; FTAR, Frontal Theta/Alpha Ratio; CDTABR, Central (Delta+Theta)/(Alpha+Beta) Ratio; CTAR, Central Theta/Alpha Ratio. Due to the inherent right-skewed distribution of EEG spectral power ratios, these specific parameters underwent natural logarithmic transformation prior to Z-score standardization to fulfill regression assumptions. All continuous variables were subsequently Z-score standardized to ensure the comparability of effect sizes. Consequently, the ORs represent the relative change in risk per one standard deviation (1-SD) increase in the respective predictor. Model 1 represents the unadjusted analysis including the core cross-modal features identified via LASSO regression. Model 2 is fully adjusted for predefined demographic covariates (age and education level). A Firth’s penalized logistic regression was employed in both models to rigorously mitigate small-sample bias and potential data separation.

To directly address potential algorithmic bias arising from the numerical imbalance between the MCI (*n* = 37) and NC (*n* = 23) groups, a *post-hoc* sensitivity analysis was conducted. We randomly undersampled the MCI group to create a strictly balanced cohort (23 MCI vs. 23 NC). When refitting the multivariable model within this balanced subset (*N* = 46), the core neurophysiological signature demonstrated favorable stability. Although the artificially reduced sample size inherently compromised statistical power and widened the confidence intervals, the directional effects of the core features remained consistent: saccadic accuracy maintained its protective trend, and the frontal/central spectral ratios (e.g., TAR, DTABR) preserved their positive predictive directionality. This balanced sub-experiment suggests that the cross-modal features identified in the full Firth’s penalized model are resilient to class imbalance and reflect genuine pathophysiological alterations.

**FIGURE 7:**
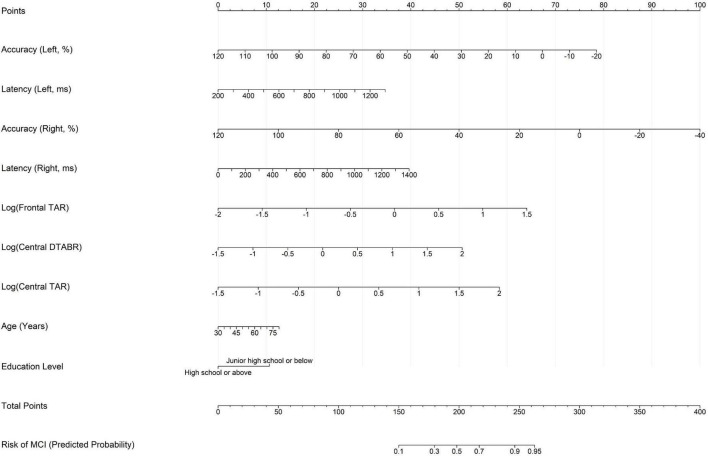
Individualized nomogram model based on cross-modal features for predicting the risk of mild cognitive impairment. In the nomogram, “Accuracy (Left)” and “Accuracy (Right)” correspond to leftward and rightward saccadic accuracy, respectively, while “Latency (Left)” and “Latency (Right)” correspond to leftward and rightward saccadic latency. TAR, Theta-to-Alpha Ratio; DTABR, (Delta+Theta)/(Alpha+Beta) Ratio.

While certain individual variables did not achieve strict statistical significance after covariate adjustment, their collective inclusion captured the complementary neurophysiological deficits of MCI, thereby enhancing the overall predictive utility of the algorithm. Based on these integrated multivariable features, a clinical nomogram was successfully developed to facilitate intuitive and individualized risk estimation for cognitive impairment (Figure 7).

**FIGURE 8:**
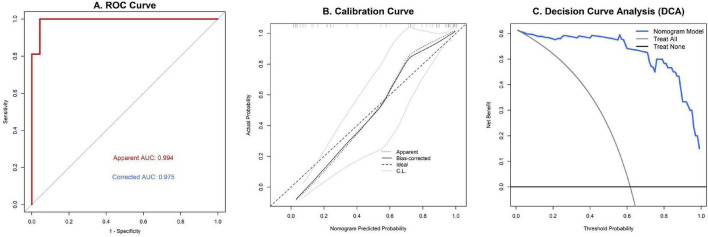
Comprehensive performance evaluation of the nomogram model based on cross-modal features for predicting MCI risk. **(A)** Receiver operating characteristic (ROC) curve showing the apparent and optimism-corrected area under the curve (AUC). **(B)** Calibration curve assessed via 1,000-bootstrap resampling, demonstrating the agreement between nomogram-predicted probabilities and actual observations. **(C)** Decision curve analysis (DCA) evaluating the net clinical benefit of the predictive model across a continuum of high-risk threshold probabilities.

The predictive performance of the multimodal nomogram was comprehensively evaluated across three dimensions: discrimination, calibration, and potential clinical utility (Figure 8). Regarding discrimination, the model demonstrated high discriminative ability with an apparent AUC of 0.994 (95% CI: 0.985–1.000). To strictly address the risk of overfitting in this modest cohort (*N* = 60), we implemented a zero-leakage validation pipeline, yielding a narrow average optimism bias of 0.019 and a bias-corrected AUC of 0.975. Furthermore, to avoid the optimism of in-sample classification, we determined the optimal clinical decision threshold using a stringent leave-one-out cross-validation (LOOCV) approach. Based on the maximum Youden’s index derived from these out-of-sample LOOCV probabilities, the optimal cut-off threshold for classifying MCI was determined to be 0.741. At this rigorously externalized threshold, the model maintained robust performance, achieving a sensitivity of 83.8% and a specificity of 91.3%. Model calibration was assessed using the bootstrap-resampled calibration curve. The analysis revealed favorable agreement between the nomogram-predicted probabilities and the actual observations, with a mean absolute error (MAE) of 0.07, indicating reliable probability estimation. Finally, Decision Curve Analysis (DCA) was conducted to determine the clinical applicability of the proposed model. Within a substantial range of threshold probabilities, the developed nomogram consistently provides greater net benefit compared to both “treat-all” and “treat-none” strategies. This suggests that the model may possess potential value as an exploratory screening tool, providing a promising proof-of-concept for future clinical translation once externally validated.

## 4 Discussion

The present study focused on an Asian middle-aged and older population and, within a unified experimental framework, integrated memory-guided saccade performance with EEG spectral features to systematically investigate cross-modal neurophysiological alterations in mild cognitive impairment. Our findings demonstrate that patients with MCI exhibited significantly reduced saccadic accuracy, prolonged latency, and diminished final saccadic gain during the MGS (Figure 2). Concurrently, EEG analyses revealed significantly elevated low-/high-frequency power ratios (DAR, DTABR, and TAR) across the frontal, parietal, temporal, occipital, and central regions, consistent with a widespread pattern of cortical spectral slowing (Figure 3).

At the feature selection level, a rigorously constructed LASSO regression model identified a cross-modal diagnostic signature comprising seven core variables: leftward and rightward saccadic accuracy, leftward and rightward latency, and slow-wave spectral indices in the frontal and central regions (Figure 6). This combined model demonstrated robust diagnostic performance in internal resampling validation (Figure 8). Although internal correction procedures were applied to mitigate optimism bias, the relatively modest sample size warrants cautious interpretation, and external validation in independent cohorts is necessary to establish generalizability.

Collectively, these findings suggest that during the MCI stage, networks subserving executive control and spatial working memory may already exhibit multidimensional functional decline, accompanied by large-scale alterations in cortical oscillatory dynamics. The correspondence between behavioral abnormalities and electrophysiological changes provides cross-modal evidence for reduced efficiency within early “cognitive–motor” network interactions.

### 4.1 Executive control and spatial memory deficits revealed by the memory-guided saccades

Eye movements represent a critical interface linking visual input to higher-order cognitive processing. Oculomotor parameters offer objective indices of sensory integration, attentional regulation, and executive control (Lin et al., 2023). Among various eye-tracking paradigms, the MGS—encompassing spatial encoding, delay-period maintenance, and voluntary motor output, has been widely regarded as a sensitive probe of frontoparietal network integrity in early MCI (Bruce and Goldberg, 1985; Hutton, 2008). The generation of voluntary saccades depends on coordinated activity between the frontoparietal executive network and subcortical structures. The dorsolateral prefrontal cortex and frontal eye fields play central roles in suppressing reflexive saccades and initiating memory-driven motor commands. The parietal eye fields contribute to spatial coordinate transformation and attentional allocation, while the superior colliculus integrates motor preparation and final output signals (Xiao et al., 2006; Chen et al., 2016; MacAskill and Anderson, 2016; Jonikaitis et al., 2023). Accordingly, the MGS paradigm provides a cognitively demanding framework for assessing the global efficiency of frontoparietal network coordination.

In the present study, prolonged bilateral latency and reduced bilateral accuracy were retained in the final LASSO model (Figure 6), suggesting parallel deterioration of executive control and spatial working memory during the MCI stage. Community-based studies have similarly reported that higher-order oculomotor parameters derived from MGS paradigms can differentiate individuals with MCI from cognitively normal older adults, with saccadic accuracy and latency emerging as relatively stable and discriminative behavioral markers (Sha et al., 2026).

From a neurobiological perspective, prolonged MGS latency may reflect reduced efficiency within prefrontal–basal ganglia circuits responsible for suppressing stimulus-driven reflexive saccades and completing motor preparation. The MGS requires active inhibition of impulsive responses during the delay period and sustained maintenance of target location. Increased latency therefore likely indicates impaired processing speed and inhibitory control, consistent with previous findings of prolonged latency in amnestic MCI during overlap saccade paradigms (Hutton, 2008). Reduced accuracy, in turn, reflects compromised performance across multiple stages, including spatial encoding, delay maintenance, and final localization output. Eye-tracking studies have shown that compared with healthy controls, individuals with MCI exhibit greater spatial dispersion during MGS. This decline in high-fidelity spatial memory maintenance may be attributable to reduced efficiency within parietal and frontal executive networks (Crawford et al., 2017).

Importantly, the LASSO model retained oculomotor features for both leftward and rightward directions, suggesting that frontoparietal dysfunction in MCI likely reflects diffuse, bilateral hemispheric network degeneration rather than focal lateralized pathology. However, in the absence of structural or functional neuroimaging data, this interpretation remains speculative and warrants confirmation in multimodal studies.

Correlation analyses further demonstrated that MMSE and MoCA scores were positively correlated with leftward and rightward saccadic accuracy (*r* = 0.49–0.55, all *P* < 0.01) and negatively correlated with slow-wave spectral indices (DAR, DTABR, and TAR; *r* = −0.29 to −0.57, all *P* < 0.01) (Figures 4, 5). These findings indicate that declining cognitive performance is accompanied by both behavioral impairment and a shift of cortical oscillatory activity toward lower frequencies. Heatmap visualization and curve-fitting analyses supported these trends, suggesting that combined MGS and EEG features not only distinguish diagnostic groups but also track functional changes along a continuous cognitive spectrum. Overall, latency and accuracy during the MGS jointly capture executive control and spatial working memory dimensions of frontoparietal function, providing direct behavioral evidence for early network degeneration in MCI.

### 4.2 EEG spectral abnormalities reflect early cortical network reorganization

By capturing synchronized oscillatory activity across large neuronal populations, EEG provides a valuable window into cortical functional states and network dynamics. Owing to its high temporal resolution, cost-effectiveness, and ease of standardization, EEG has been extensively applied in studies of cognitive impairment and neurodegenerative disease (Cullen et al., 2007; Raufi and Longo, 2022). Within conventional frequency band frameworks, alpha and beta oscillations are primarily associated with sensorimotor integration, attentional modulation, and higher-order cognitive processing. Reductions in these higher-frequency bands often indicate diminished cortical information integration or suppression of sensorimotor circuits. In contrast, increased delta and theta activity typically reflects cortical dysfunction or compensatory hypersynchronization following neural injury and has been linked to aberrant thalamocortical activation (Finnigan and van Putten, 2013; Neske, 2015; Nuñez and Buño, 2021; Prete et al., 2022).

Abnormal EEG patterns in MCI have been documented across diverse conditions. Previous studies have demonstrated that using multichannel EEG and single-channel ERP approaches, respectively, successfully identified individuals with MCI (Cejnek et al., 2021; Lee and Lee, 2025). Cejnek et al. (2021) employed novelty detection algorithms to uncover latent abnormal EEG patterns. Furthermore, Geng et al. reported elevated delta and theta power in sleep EEG recordings from patients with MCI, suggesting that spectral abnormalities persist across both wakefulness and sleep states (Geng et al., 2022). Across the Alzheimer’s disease continuum, EEG spectral changes exhibit stage-dependent evolution, allowing discrimination of disease phases based on oscillatory characteristics (Jiao et al., 2023).

At the mechanistic level, individuals across the MCI-Alzheimer’s disease spectrum typically exhibit enhanced slow-wave activity and suppressed fast oscillations, representing the canonical pattern of spectral slowing. Prior studies in Alzheimer’s disease have demonstrated significantly reduced alpha power during eyes-closed resting conditions, indicating disruption of intrinsic cortical rhythmicity (Murty et al., 2021). Concurrent increases in theta power and decreases in gamma activity further reflect imbalance in oscillatory dynamics (Meghdadi et al., 2021). Enhanced delta and theta oscillations have been associated with increased neuronal synchrony, reduced synaptic efficiency, and impaired hippocampal–cortical information integration, whereas diminished alpha and beta activity suggests reduced stability of executive and default mode networks (Jelic et al., 1998; Colgin, 2013). This framework provides a neurophysiological basis for interpreting EEG abnormalities during the MCI stage.

Consistent with the spectral slowing hypothesis, our results showed significantly elevated DAR, DTABR, and TAR values across multiple cortical regions in MCI (Figure 3). After accounting for multicollinearity in high-dimensional data, the LASSO model identified frontal TAR and central DTABR and TAR as the most diagnostically informative EEG features. These topographically specific indices contributed independent statistical value and demonstrated functional correspondence with oculomotor abnormalities.

Enhanced slow-wave activity in the frontal region is consistent with reduced efficiency of prefrontal-thalamic information integration. As a central hub of executive control and spatial working memory, the prefrontal cortex exhibits increased low-frequency oscillatory activity when inhibitory control and cognitive planning functions are compromised. Previous studies have reported significant associations between elevated frontal theta activity and executive and working memory deficits in MCI (Tseriotis et al., 2024; Noh et al., 2026). Abnormal spectral rhythms in the central region suggest early functional reorganization within the sensorimotor system. The central region encompasses the primary sensorimotor cortex, a key node in motor planning and execution. Studies combining transcranial magnetic stimulation and EEG have shown that sensorimotor network connectivity may already be altered during early cognitive impairment, even in the absence of overt motor symptoms (Nardone et al., 2021). In the present study, increased central DTABR and TAR reflected coexisting enhancement of low-frequency activity and suppression of higher-frequency rhythms. Such oscillatory reconfiguration may represent reduced activation efficiency within motor execution networks, providing a mechanistic explanation for the significantly prolonged latencies for both leftward and rightward saccades observed during the MGS.

At a broader systems level, successful voluntary eye movements require precise coordination between prefrontal–parietal planning circuits and sensorimotor execution systems (McDowell et al., 2008). The concentration of core EEG features within frontal and central regions suggests that both cognitive planning and motor execution circuits may be concurrently affected at the cross-modal level. On the one hand, reduced prefrontal network efficiency compromises spatial information maintenance and inhibitory control (Jiang et al., 2024), contributing directly to decreased saccadic accuracy. On the other hand, diminished activation efficiency within the primary sensorimotor cortex delays motor output processes (Ferreri et al., 2016), contributing to prolonged latency. The functional convergence of these impairments results in the dual deficit, reduced accuracy and increased latency observed in the MGS.

Taken together, the structured associations between frontal and central spectral abnormalities and MGS behavioral metrics suggest that, even in early MCI, the efficiency of coordination between cognitive planning and motor execution networks may already be diminished, reflecting a degree of “cognitive–motor” decoupling. This cross-network oscillatory reorganization provides novel electrophysiological evidence for the system-level nature of functional impairment in MCI.

### 4.3 Clinical translational potential and advantages of multimodal feature integration

In current clinical practice, early screening for mild cognitive impairment still relies heavily on subjective cognitive scales, such as the Mini-Mental State Examination and the Montreal Cognitive Assessment (MoCA). Performance on these instruments is susceptible to the influence of educational attainment and cultural background. Although cerebrospinal fluid biomarkers and molecular imaging provide high pathological specificity for Alzheimer’s disease, their invasiveness, high cost, and exposure to radioactivity limit their feasibility for large-scale implementation in community-based populations.

The present study identified early neurofunctional alterations in individuals with MCI from both behavioral and electrophysiological perspectives. At the behavioral level, patients with MCI demonstrated reduced accuracies and prolonged latencies for both leftward and rightward saccades during the memory-guided saccades. At the electrophysiological level, widespread cortical spectral slowing was observed, characterized by a shift toward lower-frequency oscillatory activity.

The core features selected by the LASSO model were concentrated in frontal and central spectral indices, together with oculomotor behavioral metrics, suggesting that cross-modal integration enhances discriminative performance. Notably, parietal features were not retained in the final model. This does not imply that parietal function remains unaffected; rather, it likely reflects the intrinsic property of the LASSO algorithm to select variables with the highest independent informational value under conditions of multicollinearity. Within highly synchronized cortical networks, frontal and central spectral indices may carry the greatest independent informational entropy. As representative network hubs, these regions effectively capture global oscillatory degeneration within the broader frontoparietal–sensorimotor executive network.

The coordinated decline in cognitive and motor function at the network level may represent a manifestation of impaired “cognitive–motor integration,” a phenomenon increasingly recognized in the early stages of central nervous system degeneration. From a translational perspective, the MGS–EEG cross-modal model established in this study demonstrated robust diagnostic stability following rigorous internal validation (Figure 8). Importantly, the resulting individualized nomogram (Figure 7) and the seven extracted features are characterized by low cost, non-invasiveness, and strong neurobiological interpretability. These findings provide an early conceptual foundation and potential targets for the future exploration of community-oriented digital neuropsychological screening paradigms. However, we emphasize that this model is strictly exploratory and is not yet suitable for direct clinical implementation or deployment at this stage. Indeed, as highlighted by recent comprehensive methodological reviews, translating computational and AI-driven diagnostic frameworks into real-world clinical practice entails substantial generalizability and validation challenges (Rasool et al., 2026).

### 4.4 Limitations and future directions

Despite the translational promise of the proposed cross-modal diagnostic model, several limitations warrant consideration. First, due to stringent inclusion criteria and the practical challenges associated with clinical data acquisition, the sample size was relatively modest. Specifically, despite implementing LASSO to rigorously reduce dimensionality, the final multivariable model operated under a constrained events-per-variable (EPV) ratio. We explicitly acknowledge that this falls below the traditional epidemiological threshold of 10–20 required for classical logistic regression stability. While we implemented advanced mathematical safeguards, specifically utilizing Firth’s penalized maximum likelihood estimation to robustly counteract low-EPV parameter estimation bias, alongside 1,000 bootstrap resamples for optimism correction, we recognize that algorithmic penalization cannot fully substitute for larger clinical cohorts. Consequently, the current nomogram cannot be recommended for clinical deployment at this stage; rather, it serves strictly as an exploratory, hypothesis-generating proof-of-concept. Furthermore, we acknowledge that certain variables within our model, specifically rightward saccadic latency (selected in 47.6% of bootstrap iterations), exhibited moderate selection stability. The inclusion of these features reflects the inherent complexity of identifying robust biomarkers in modest clinical cohorts. While such features must be interpreted with caution, their collective integration—rigorously tempered by Firth’s penalization—allows for a more comprehensive capture of the heterogeneous neurophysiological deficits in MCI.

Second, the absence of gold-standard pathological biomarkers (e.g., CSF amyloid/tau assays or PET imaging) precluded the etiopathogenic classification of our cohort. Clinical MCI is a highly heterogeneous syndrome driven by diverse underlying pathologies, including early Alzheimer’s disease, cerebrovascular disease, and mixed neurodegenerative processes (Jack et al., 2018). Consequently, the observed MGS-EEG abnormalities reflect the large-scale functional uncoupling characteristic of the clinical MCI syndrome, rather than a specific neurodegenerative etiology. It must be explicitly emphasized that the multimodal framework proposed in this study provides diagnostic evidence strictly for clinical MCI, and these findings cannot be specifically attributed to Alzheimer’s-related cognitive impairment.

Third, the EEG methodology employed in this study presents inherent limitations. The analysis was based on a 32-channel system and a 30-s artifact-free segment. Segment length fundamentally influences spectral characteristics: shorter segments yield fewer sub-epochs for averaging, inherently increasing the statistical variance of the power estimates, whereas excessively long segments violate the assumption of signal stationarity by capturing unobserved shifts in vigilance. Therefore, while the chosen 30-s window represents an optimal trade-off to ensure signal stationarity and minimize state transitions in a cognitively impaired cohort, it may exhibit higher spectral variance compared to longer recordings. Furthermore, the low spatial resolution of a 32-channel clinical montage precludes precise source localization. Consequently, our macro-scale regional analyses provide limited capabilities for resolving deep cortical generators or elucidating precise neuroanatomical network mechanisms underlying the observed cognitive-motor uncoupling.

Fourth, while our predictive model was stringently adjusted for demographic covariates (age and education), we were unable to simultaneously control for vascular risk factors (e.g., hypertension, hyperlipidemia) due to sample size constraints. Given that covert cerebrovascular burden can independently accelerate cortical spectral slowing and oculomotor decline, the potential confounding influence of these vascular factors remains a limitation that must be addressed in future research.

Fifth, our behavioral assessment relied exclusively on a single oculomotor paradigm. Because MGS is a composite task requiring the simultaneous recruitment of spatial working memory, inhibitory control, and selective attention, we cannot definitively disentangle the independent contributions of these specific cognitive domains to the observed oculomotor deficits.

Sixth, the cross-sectional design of the present study precluded the calculation of cohort-specific test-retest reliability metrics (such as the Intraclass Correlation Coefficient, ICC) for the MGS parameters. Although existing oculomotor literature consistently demonstrates good to excellent temporal stability for saccadic latency and spatial accuracy in aging and cognitively impaired populations (ICC typically > 0.70) (Blekher et al., 2009), the absence of within-cohort repeated measures limits our ability to definitively isolate stable neurophysiological trait differences from transient measurement noise.

To systematically address these limitations, future longitudinal investigations must leverage large-scale, multicenter cohorts to establish the intra-subject reliability of these behavioral biomarkers and evaluate the prospective utility of this combined framework in predicting rates of cognitive decline. Integration with high-resolution functional magnetic resonance imaging and peripheral plasma biomarkers will further elucidate the molecular and structural network mechanisms underlying the observed “cognitive–motor” decoupling. Behaviorally, incorporating a broader battery of targeted oculomotor paradigms—such as the antisaccade task to specifically isolate inhibitory control—will be essential to parse the distinct cognitive components driving early oculomotor dysfunction. Methodologically, analyzing such complex, large-scale multimodal datasets will require transitioning from traditional penalized regression to advanced deep learning architectures. Recent algorithmic innovations—including explainable artificial intelligence (NeuroXAI) frameworks for neurodegenerative disease detection (Bunterngchit et al., 2025), biologically informed hybrid attention models (Aslam et al., 2026), and deep neurocomputational fusion strategies for multi-domain EEG analysis (Rasool et al., 2025), offer powerful tools for decoding highly non-linear neurophysiological patterns. Integrating these cutting-edge computational approaches with our behavioral-electrophysiological paradigm will be critical for advancing this exploratory framework toward precision clinical diagnostics.

## 5 Conclusion

Within a unified experimental framework, this exploratory study established a preliminary screening model for MCI that integrates memory-guided saccadic parameters with EEG spectral features. By employing LASSO regression coupled with Firth’s penalized estimation to robustly mitigate small-sample bias, we identified a seven-feature neurophysiological signature which, when integrated with demographic covariates, formed a predictive framework that achieved robust discriminative performance. We explicitly acknowledge that these findings serve as a hypothesis-generating proof-of-concept and require future external validation in larger, multi-center cohorts. Nevertheless, this multimodal approach provides a promising conceptual foundation for the development of non-invasive, community-based digital screening tools for early cognitive impairment.

## Data Availability

The raw data supporting the conclusions of this article will be made available by the authors, without undue reservation.
